# PRP significantly promotes the adhesion and migration of vascular smooth muscle cells on stent material

**DOI:** 10.1186/s40001-023-01541-5

**Published:** 2023-12-09

**Authors:** Yin-Di Wu, Hong-Jing Jiang, Hao-Hao Zhou, Jian-Yi Xu, Qing Liu, Xu-Heng Sun, Yue-Heng Wu, Zhan-Yi Lin

**Affiliations:** 1 School of Medicine, South China University of Technology, Guangzhou, 510006 Guangdong China; 2 Guangdong Provincial People’s Hospital (Guangdong Academy of Medical Sciences, Guangdong Academy of Medical Sciences), South Medical University, Guangzhou, 510080 Guangdong China; 3 Ji Hua Institute of Biomedical Engineering Technology, Ji Hua Laboratory, Foshan, 528200 Guangdong China; 4 School of Biological Sciences and Engineering, South China University of Technology, Guangzhou, 510006 Guangdong China

**Keywords:** Platelet-rich plasma (PRP), Smooth muscle cell (SMC), Cell migration assays, Decellularized extracellular matrix (ECM)

## Abstract

**Background:**

The adhesion and survival state of cells on scaffold material is a major problem in tissue-engineered blood vessel (TEBV) culture. Platelet-rich plasma (PRP) contains a large amount of biologically active factors and fibrin, which is expected to play an important role in TEBV culture.

**Purpose:**

To combine PRP with cells and scaffold material to promote cell adhesion and biological activity on the scaffold material.

**Methods:**

The adhesion status and migration of SMCs under the optimal concentration suitable for SMC growth and the optimal concentration of PRP were examined by scanning electron microscopy, HE staining, CCK-8 assays, qPCR, WB, and other experimental methods and compared with those under the conventional culture (20% FBS); finally, the effect of PRP on the deposition of ECM in vascular tissue engineering culture was verified by three-dimensional culture.

**Results:**

PRP at 20% is a suitable concentration for SMCs. Compared with the control group, the 20% PRP group had better migration, and the number of SMC adhesions was significantly higher than that of the control group. In addition, collagen deposition in the experimental group was significantly higher than that in the control group.

**Conclusion:**

PRP** (**20%) can promote SMC adhesion, migration, and collagen deposition on the scaffold material.

## Introduction

The culture of tissue-engineered blood vessels consists of three basic elements: an adequate amount of seed cells, a suitable scaffold material, and a complex culture environment [1, 2]. Due to the long culture time, the adhesion status of seed cells on top of the scaffold material is directly related to the outcome of TEBV culture. PGA material is a biodegradable polymer hydrophobic material that can develop a porous three-dimensional structure through nonwoven technology [3]. We found that in the traditional method for TEBV culture, adhesion of smooth muscle cells on the nonwoven PGA material adhesion is poor, cells in an uncontrollable proportion fall on the bottom of the reactor or fall on the bottom of the current technology and cannot be completely removed, and part of the residual seed cells will be in the bottom and cocultured with TEBVs. On the one hand, the cells will remove the upper part of the TEBV nutrients. On the other hand, the bottom cells die when they reach a certain density, and it is not known whether some factors will be released in this process to have uncontrollable effects on the upper TEBV layer.

To make the culture process smoother and to improve the biological activity of seeded cells on the scaffold material, many scholars have tried various methods [4], and some scholars combined gelatin and PLLA material and found that it could improve cell adhesion and migration on the material. LI electrospun keratin and PCL material were used to form a composite vascular tissue engineering scaffold material, and this biocomposite scaffold enhanced endothelial cell (EC) adhesion and growth [5]. Some researchers have also enhanced cell adhesion on top of the material by adding collagen or both fibrin and gelatin [6, 7]. These methods modify the properties of the material by altering its surface morphology and characteristics, but the techniques are more complex.

Growth factors play an important role in biological activities, such as cell adhesion, migration, secretion, and proliferation [8, 9]. Platelet-rich plasma (PRP) contains a variety of growth factor components, such as TGF, VEGF, and PDGF. PRP also contains fibronectin, a three-dimensional meshwork structure that can effectively store a variety of growth factors and cells [10]. PRP is widely available, easy to obtain, and commercially available. It can be obtained repeatedly and multiple times with minimal trauma [11–13] and is already available in established commercial products. We promoted the adhesion and migration of SMCs on top of the scaffold material by adding a certain concentration of PRP to the smooth muscle cell suspension and then placing it onto the PGA scaffold.

## Materials and methods

All experiments were conducted in accordance with the ethical regulations of Guangdong Provincial People's Hospital, Guangdong Academy of Medical Sciences, and the ethics number is No. GDREC2019285h (RI).

Nonwoven PGA material was purchased from the United States (Biomedical Structures, Rhode Island, Warwick, USA), PRP was obtained from six healthy volunteers (aged 25–31 years), all with informed signed consent, and smooth muscle cells were obtained from the residual human aortic tissue of a heart transplant donor.

### Preparation of PRP

The two-step centrifugation method was used to obtain PRP. First, venous blood was taken with anticoagulation tubes containing sodium citrate (KWS, China), and two-step centrifugation was performed at 200 g*15 min and 400 g*15 min to collect PRP and PPP components. Whole blood and PRP were subjected to platelet counting, so that the platelet concentration of PRP was four-to-six times that of whole blood [14, 15]. Platelet markers were stained using the Richter (Biosharp, China) staining method. The PRP was stored at − 80 °C for 1 h and then thawed at room temperature for 1 h. The procedure was repeated three times, and the PRP solution without cells was obtained by centrifugation for the following experiments and stored at − 80 °C [15].

### Isolation and identification of SMCs

Human aortic tissue samples of 2 cm*2 cm were used for primary culture of smooth muscle cells by the applanation culture method. Briefly, the mesangial tissues were first evenly attached to a T25 culture flask (Corning, USA) and incubated in a 37 °C incubator (Thermo, USA) containing 5% CO2, and 4–5 ml of DMEM/F12 (Dulbecco’s Modified Eagle Medium, Corning, USA) medium containing 20% FBS (Fetal Bovine Serum, Gibco, USA) was added [16]. After 4 h, the flask was not shaken for 1 week to prevent the tissue from floating and the cells from climbing out. After 2 weeks, the cells were passaged until the P3 generation, 5*10^4 cells were collected on cell crawlers (Biosharp, China), immunofluorescence staining was performed according to the instructions, and the cells were treated with antibody (calponin, smoothelin, 1:500, Abcam, USA) and DAPI working solution (Solarbio, China). Then, the samples were processed and examined microscopically [17].

### Electron microscopic detection

PGA materials from different treatment groups (A: control group treated with 20% FBS, B: experimental group treated with 20% PRP, C: control group with smooth muscle cells, and D: experimental group with smooth muscle cells) were fixed with 3 ml of 2.5% glutaraldehyde, the samples were rinsed in 0.1 mol/L sodium dimethylcarbamate buffer (pH 7.4), and the PGA materials were placed in buffer solution at 4 °C overnight. Twenty-four hours later, the buffer solution was removed, and the PGA materials were soaked in 1% citric acid for 1 h. The PGA materials were repeatedly rinsed with the buffer solution. After that, the PGA material was subjected to gradient dehydration treatment and finally treated with isoamyl acetate alternate. Observations were performed with a scanning electron microscope (S-3500N, Japan) [18].

### Growth factor release assay

To determine the ability and time of PRP to release growth factors on top of PGA material, we used PDGF and TGF-β1 as assay indicators. The above nonwoven PGA material was used at 1 cm*1 cm, and PRP was preconfigured into 20% PRP solution with DMEM/F12. One hundred microliters of 20% PRP solution was taken and injected dropwise onto the PGA material, fixed with a cotton swab, and placed in a 24-well plate (Corning, USA) for incubation, and 1 ml of PBS solution was added to each well. The medium was collected at different time points and placed at − 80 °C. The medium was stored at − 80 °C for 3, 6, 9, 12, 15, 18, 21, 24, 27, and 30 days according to the instructions (DuoSet®; R&D Systems, Minneapolis, MN, USA), and the concentrations of PDGF and TGF-β1 released from the assay material were quantified by ELISAs.

### CCK-8 experiment

SMCs in different groups (20% FBS, 10% PRP, 20% PRP, 30% PRP, 40% PRP, 50% PRP, and 100% PRP) were inoculated in 96-well plates (*n* = 6) at a density of 3000 cells/well for 1, 3, 5, 7, and 9 days, and 10% CCK-8 reagent was added according to the CCK-8 kit (Dojindo, Japan) and incubated for 2 h at 37 °C. Then, the absorbance of the medium at 450 nm was measured by enzyme marker and compared [19].

### Cell scratch experiments

SMCs in different groups (20% FBS, 20% PRP) were plated in 6-well plates at 10*10^4^ cells/well (*n* = 6), and the cells were laterally scored in the middle of the well plate with a 20 µl gun tip and photographed at 0–6–12–18 h. The scored area was calculated using ImageJ [20].

### Quantitative real-time PCR

SMCs in different groups (20% FBS, 20% PRP) were plated in 6-well plates at 10*10^4^ cells/well (*n* = 3) and incubated in DMEM with 20% FBS for 3 days. Then, total RNA was extracted using the TRIzol kit (TaKaRa, Japan) according to the instructions, total RNA was reverse transcribed using the reverse transcription kit (TaKaRa, Japan), and the cDNA obtained by reverse transcription was used with SYBR Premix Ex Taq (TaKaRa, Japan) according to the instructions [11]. The conditions for real-time PCR were 40 cycles at 95 °C for 10 s and 60 °C for 30 s, and GAPDH was used as a reference. The following is the base pair composition of the primers used (Table 1):

**Table 1:** Real-time PCR primers and product sizes

Gene		Sequence (5′-3′)	Size (bp)
GSK3B	Sense	TATGGTCTGCTGGCTGTGTG	120
	Antisense	GCTCCCTTGTTGGAGTTCCC	
CTNNB1	Sense	GTACCGGAGCCCTTCACATC	165
	Antisense	CAGCTTCCTTGTCCTGAGCA	
MMP2	Sense	AGGATGGCAAGTACGGCTTC	185
	Antisense	CTTCTTGTCGCGGTCGTAGT	
MMP9	Sense	GGACAAGCTCTTCGGCTTCT	126
	Antisense	TCGCTGGTACAGGTCGAGTA	
GAPDH	Sense	TTCGTCATGGGTGTGAACCA	170
	Antisense	GTCTTCTGGGTGGCAGTGAT	

### Western blot (WB) experiment

SMC processing was performed as above. Prior to the start of the WB experiments, proteins were lysed using a protein extraction kit containing protease inhibitors and RIPA (Solarbio, China), and the concentration of total protein was detected using a BCA kit (Biosharp, China) according to the kit’s instructions [21]. The spiked volume was calculated in advance, the samples were added to 12% SDS polyacrylamide gels, keeping the concentration of the spiked samples consistent, and the total volume of the spiked samples did not exceed 25 µl. Electrophoresis was performed at a voltage of 100–120 V for 40–60 min, followed by removal of the upper part of the dentate gel and the lower part of the excess gel components, and the gel was transferred to a polyvinylidene difluoride membrane (Millipore) at 140–160 V for 30 min, followed by incubation in 5% skim milk in Tris-buffered saline containing 0.05% and 1% Tween 20 (TBST) for blocking. Then, the membrane was incubated with the following primary antibodies: anti-CNN (1:500; Abcam, USA), anti-SMTN (1:2000; Abcam, USA), anti-GSK-3β (1:1000; Abcam, USA), anti-p-GSK-3β (1:500; Abcam, USA), anti-catenin (1:500; Abcam, USA), anti-COL I (1:1000; Abcam, USA), anti-MMP2 (1:500; Abcam, USA), anti-GAPDH (1:10,000, Abcam, USA), and anti-Histone (1:10,000; Abcam, USA). Finally, the Millipore membrane was subjected to chemiluminescence detection using ECL protein blotting substrate (Pierce). Chemiluminescence detection was performed, followed by development of the film using an exposure machine (Tanon, Shanghai, China).

### Three-dimensional culture and Masson staining experiments

The PGA material was processed into 1 cm*1.95 cm squares, soaked in 1 M NaOH solution alkaline for 1 min, and rinsed in sterile water three times for 5 min each time. Then, the PGA material was sutured on wooden sticks containing silicone tubes with 6–0 sterile sutures and sterilized by ethylene oxide, and cell inoculation was performed on the PGA material the next day [22], with a cell density of 1*10^7^ cells/ml. The assembled material was cultured in DMEM/F12 containing 20% fetal bovine serum for 4 weeks, and the vascular matrix material was removed after 4 weeks for WB experiments and Masson staining [23]. Masson trichrome staining was performed according to the instructions (PhyEasy, China) and can be briefly summarized as sectioning, dewaxing, Masson trichrome staining, dehydration, transparency, and sealing. Collagen was stained blue.

### Statistical analysis

All reported values were averaged (*n* = 6) and are expressed as the mean ± standard deviation (SD), and significant differences were determined with a two-sample t test assuming equal variances. A value of *p* < 0.05 was considered statistically significant.

## Results

**Fig. 1:**
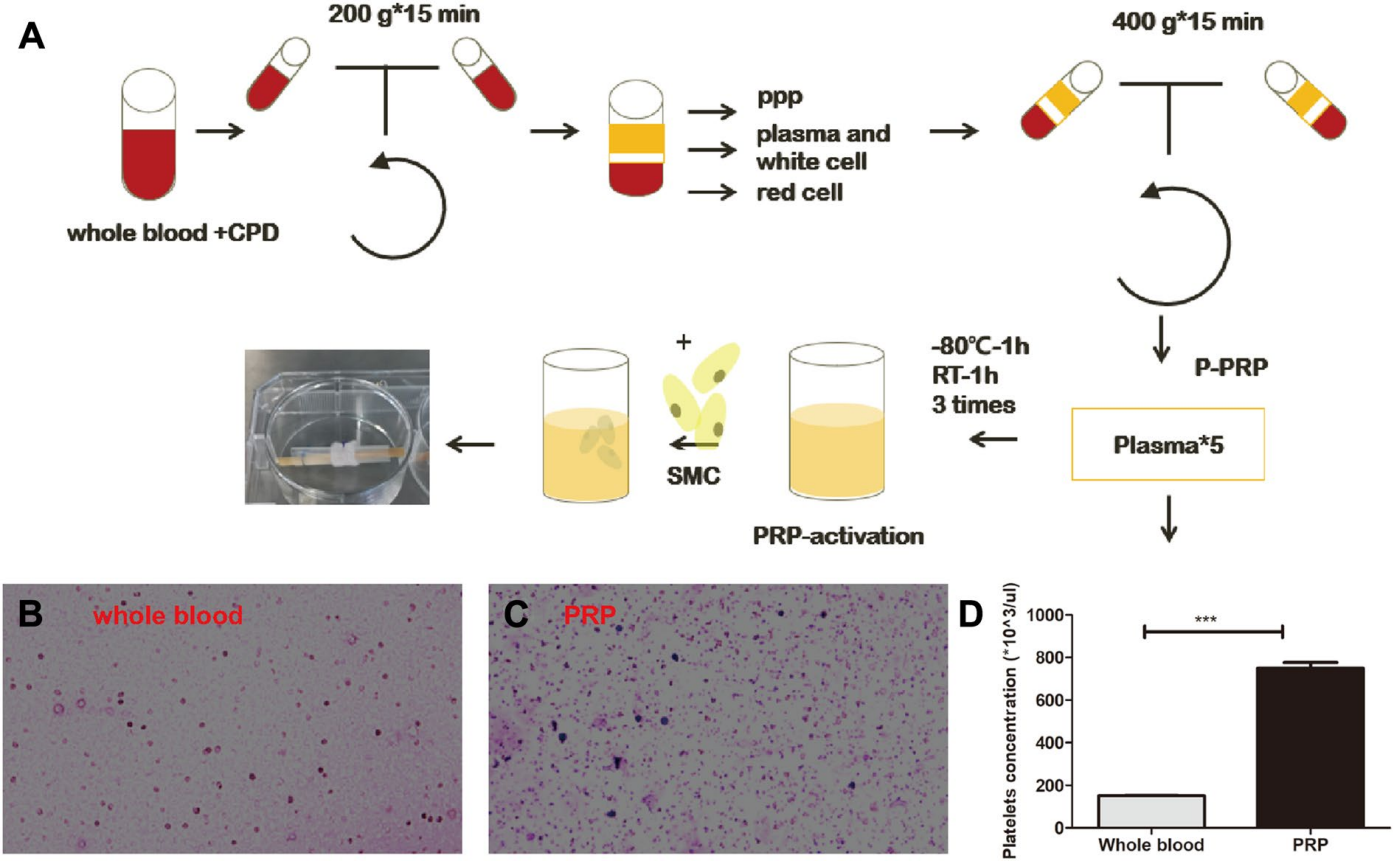
Schematic diagram of the process of PRP treatment and application, diagram of the process of PRP from treatment to use (**A**), graphs of Richter staining of whole blood and PRP (**B**, **C**), bar graphs of platelet count statistics (**D**), and comparison with the blank group ***p < 0.001. All scale bars = 50 µm

Fig. 1 A shows a schematic diagram of the production and application of PRP. A large number of platelet particles stained blue were observed on the PRP smear by Richter's staining (Fig. 1 B, C). By selecting five different fields of view for platelet counting, we found that the number of PRP platelets was 4.96 times higher than that of whole blood (Fig. 1 D), which was consistent with the preparation criteria of PRP

**Fig. 2:**
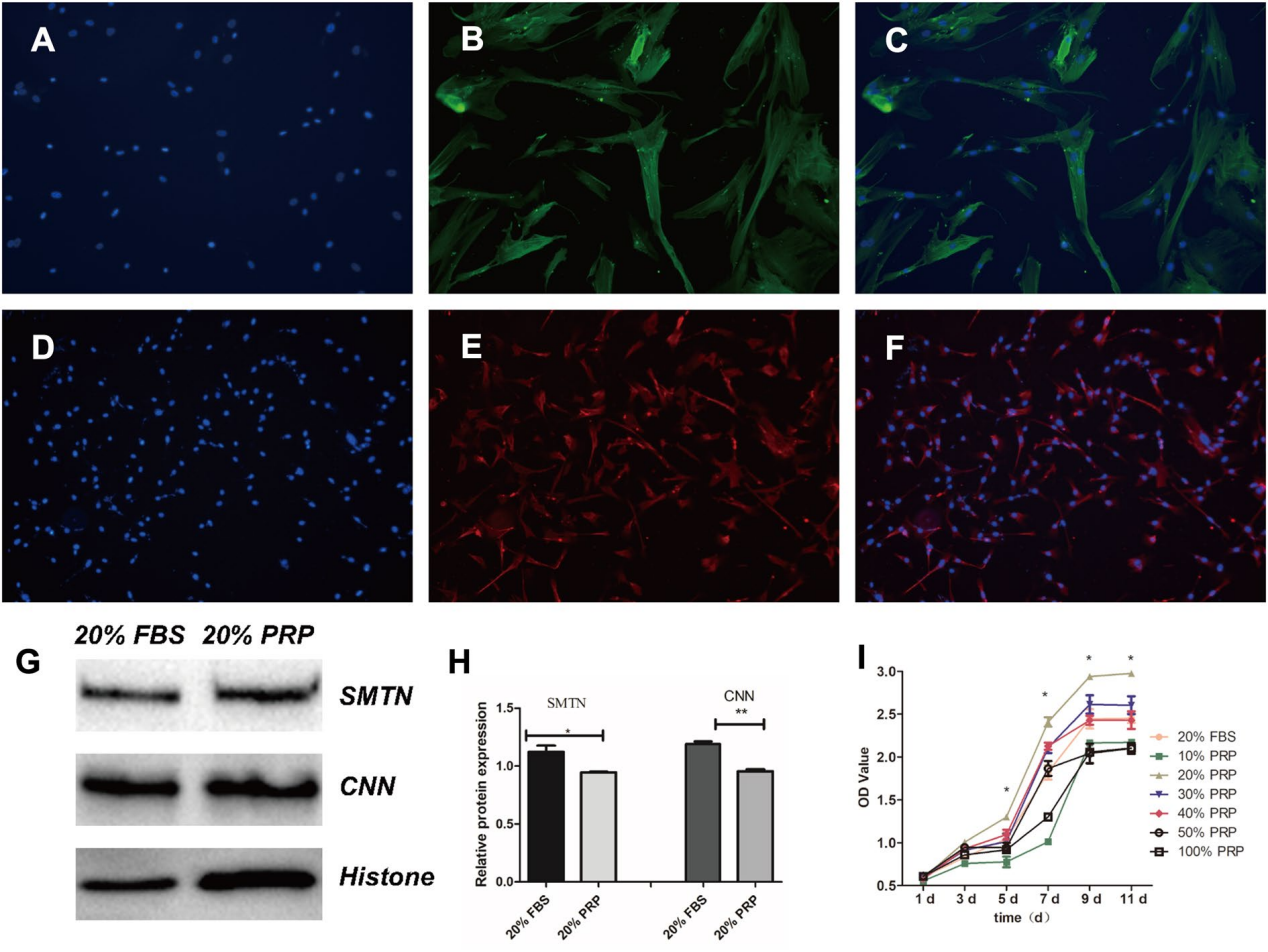
Graphs of cell phenotypic identification and results of CCK-8 experiments and graphs of cellular immunofluorescence results in the control and experimental groups (**A**–**F**). Graphs of calponin and smoothelin WB assays and their grayscale value measurements. **G**, **H** Graphs of the effect of different PRP concentrations on the proliferative activity of smooth muscle cells by CCK-8 assays (**I**). *p < 0.05, **p < 0.01, **p < 0.001 compared to the blank group. All scale bars = 50 µm

The optimal concentration of PRP for use was tested by a CCK-8 assay, and smooth muscle cell phenotypes were identified in cells from the control and experimental groups. Calponin and smoothelin are markers of contractile phenotypes of smooth muscle cells, and the phenotypic expression of both types in smooth muscle cells was found to be close to 100% by immunofluorescence analysis (Fig. 2A–G). The CCK-8 results showed that 20%, 30%, and 40% PRP all had a pro-proliferative effect on SMCs, with the 20% PRP group showing the most prominent pro-proliferative effect, and the differences were significantly different at days 5, 7, 9, and 11 (Fig. 2J). Therefore, subsequent experiments were conducted using 20% PRP for the experimental group and 20% FBS for the control group for subsequent experimental studies. WB experiments revealed that the cells used in both the experimental and control groups expressed the calponin and smoothelin proteins (Fig. 2H, I).

**Fig. 3:**
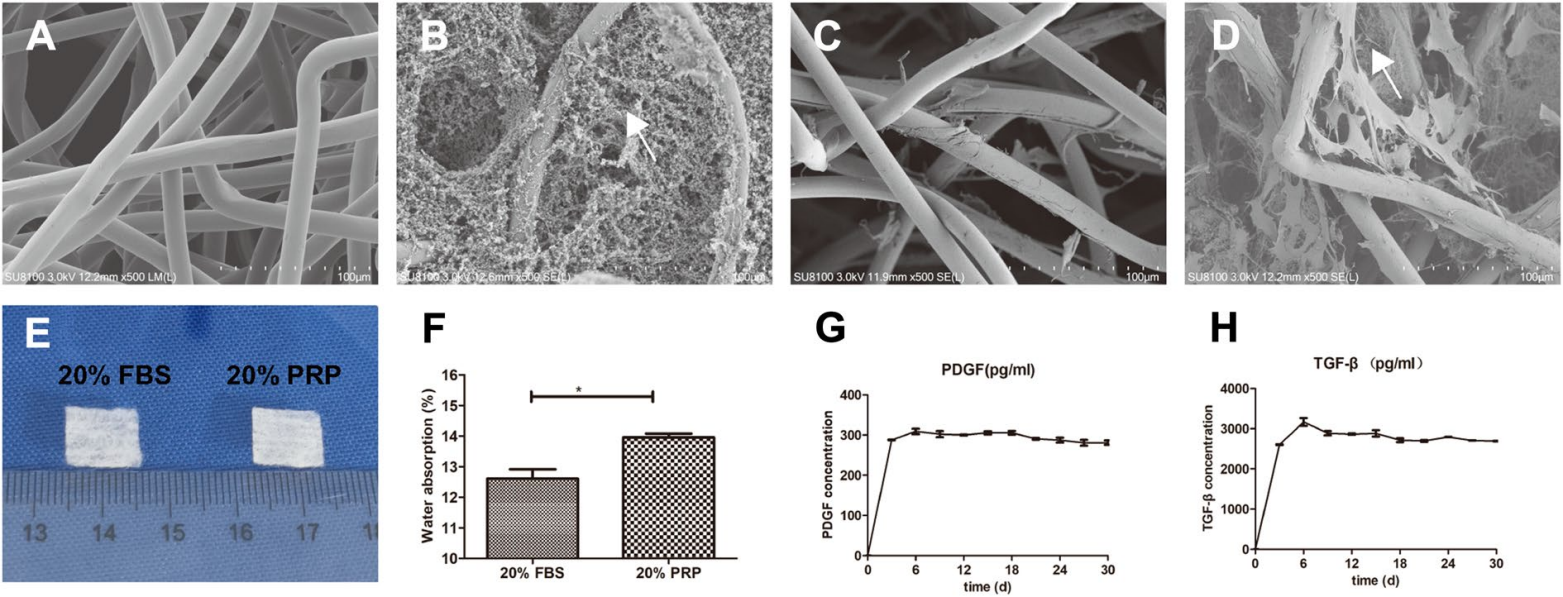
Electron microscopic detection plots, bulk plots, and growth factor release curves over time for PGA materials in the control and experimental groups. Electron micrographs of different PGA treatments (**A**, **B**), electron micrographs of cells treated with different PGA treatments (**C**, **D**), PDGF, and TGF-β after PRP treatment with PGA material for 1 month [release graphs (**G**, **H**)]. → Indicates the location of fibrin, *p < 0.05 compared to the blank group, ***p < 0.001. All scale bars = 100 µm

Electron microscopy showed that the PGA material in the PRP group contains a large number of loose fibrin filaments in the middle, as shown by the arrows, compared with that of the control group (Fig. 3B). The cells in the control group adhered to the PGA scaffold, the PGA gap was due to the large pores, a small number of cells attached to the PGA filaments, the cells attached to the PGA filaments had a single morphology, and fewer tentacles were tightly attached to the PGA filaments. In general, the number of smooth muscle cells visible in the field of view was significantly less than that of the experimental group (Fig. 3A, C), and in the experimental group, due to the presence of fibronectin, smooth muscle cells could be seen in addition to the PGA filaments, and smooth muscle cells were also observed between the PGA pores attached to fibronectin, with a variety of cell morphologies and wide tentacles (Fig. 3D). Figure 3E shows a general view of PGA, which is usually a multihollow mesh structure; the water absorption of the two materials was measured, and the PRP group showed significantly more absorption than the control group (Fig. 3F). The release of growth factors was found to be sustained for one month after PRP treatment (Fig. 3G, H).

**Fig. 4:**
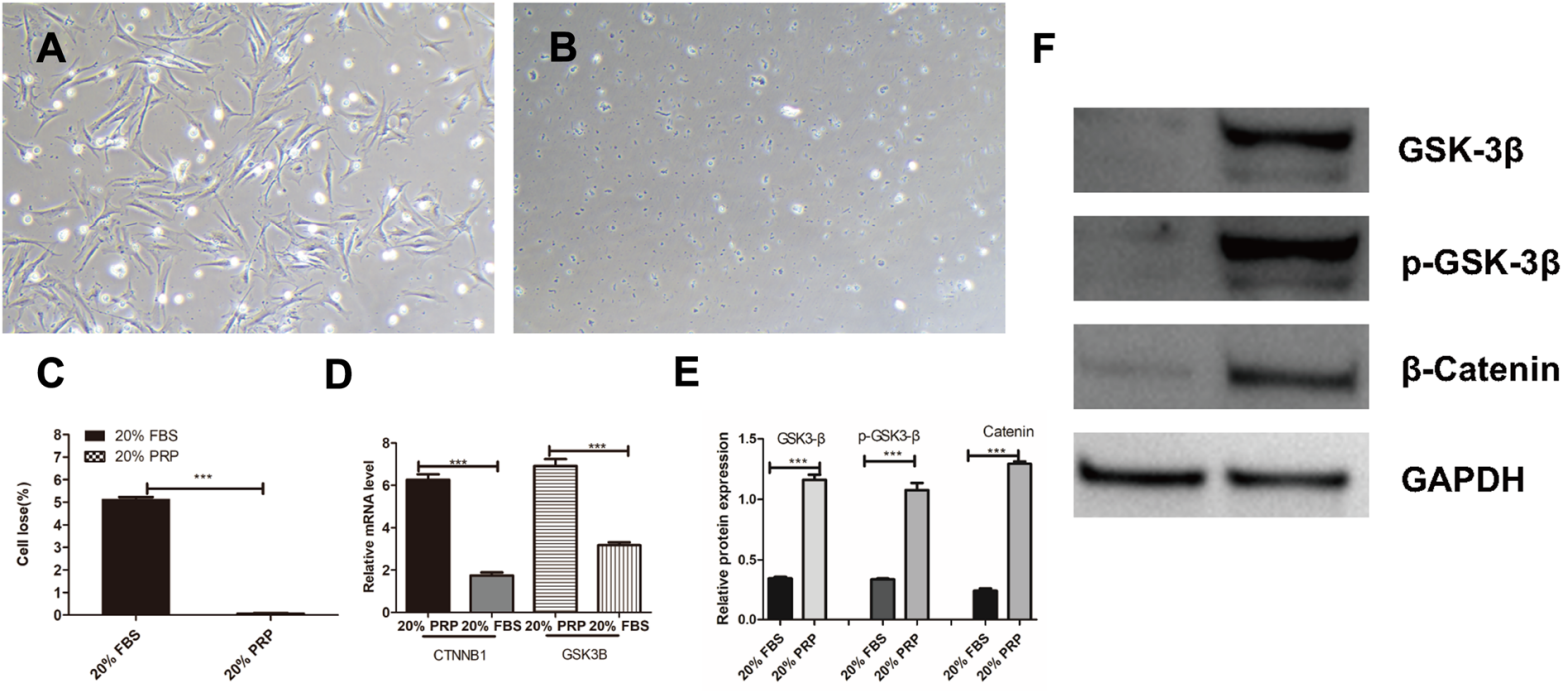
Cell adhesion assay, cell loss in the control and experimental groups (**A**–**C**), qPCR and WB assays for the CTNNB1 and GSK3β genes, and their regulatory proteins GSK-3β and β-catenin (**D**–**F**). **p < 0.01 vs. the blank group, ***p < 0.001. All scale bars = 100 µm

The results of cell adhesion experiments showed that the PGA material treated with PRP had better adsorption to smooth muscle cells, and the observation of dropped cells at the bottom after inoculation and resting revealed that the control group had more dropped cells at the bottom, reaching 5% of the inoculation number, while almost no dropped cells were observed at the bottom of the experimental group (Fig. 4A–C). The contents of the genes CTNNB1 and GSK3β and their regulatory proteins GSK-3β and β-catenin were detected by qPCR and WB analyses, and it was found that the gene expression was upregulated in the experimental group, and the protein content of both P-GSK-3β and β-catenin increased. The results indicated that PRP also promoted the protein expression of GSK-3β-activated β-catenin by upregulating adhesion-related genes, thereby promoting cell adhesion (Fig. 4D–F).

**Fig. 5:**
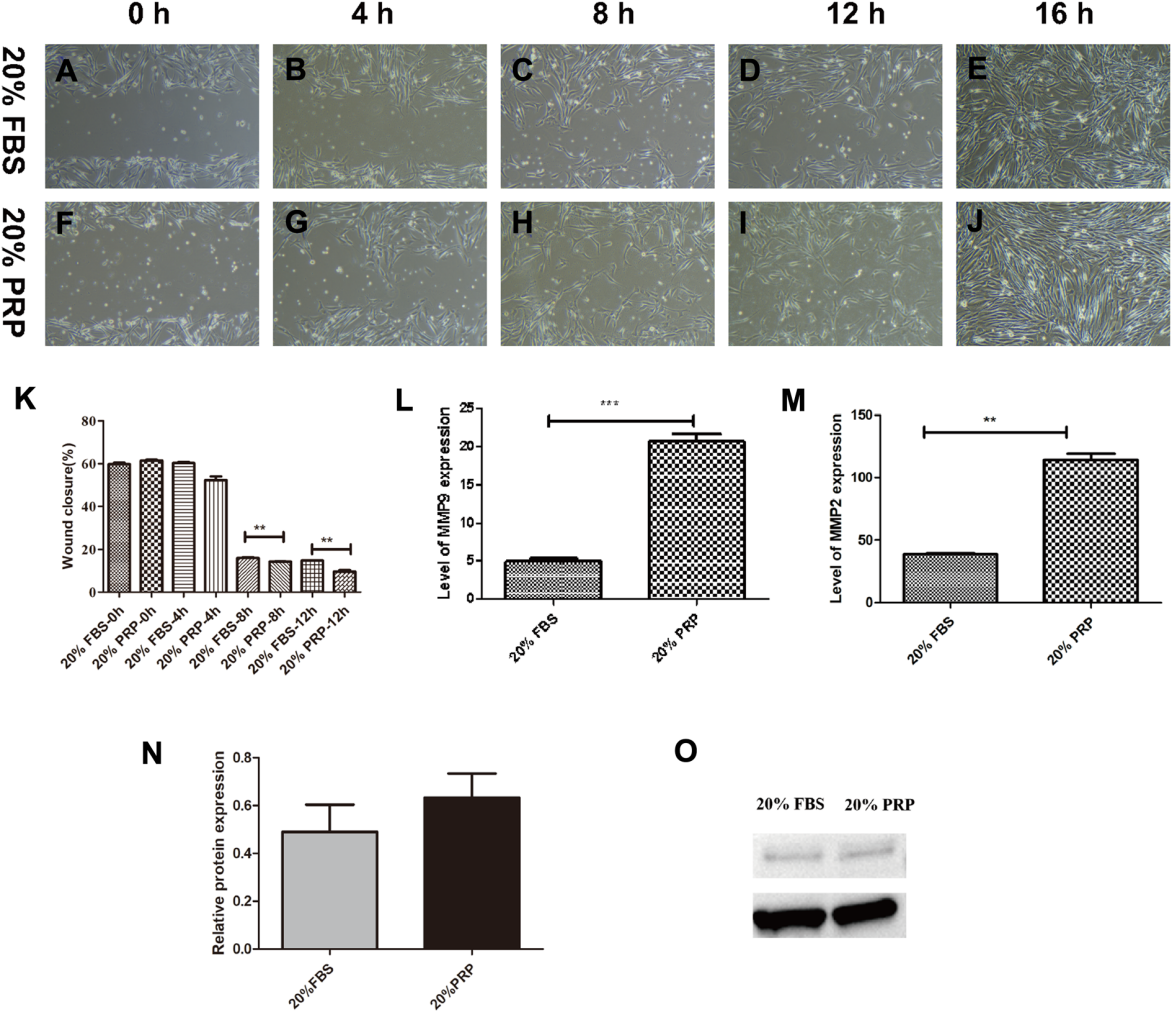
Graphs of the cell scratch assay and gene expression of MMP2 and MMP9, comparative graphs of the cell migration of the control and experimental groups (**A**‒**K**), and graphs of the cell migration-related gene expression of MMP-2 and MMP-9 (**L**, **M**). Western blot assay to determine the total MMP2 expression (**N**, **O**). **p < 0.01 vs. the blank group, ***p < 0.001. All scale bars = 100 µm

The results of the cell scratch assay showed that there was no significant difference in the cell migration rate between the experimental and control groups at hour 4. At hour 8, the cell migration rate was faster in the PRP group, and the difference was statistically significant (Fig. 5A‒K). qPCR showed that the cell migration-related genes MMP-2 and MMP-9 were both upregulated in the experimental group versus the control group (Fig. 5L, M). There was no difference in the expression of MMP2 protein (Fig. 5N, O).

**Fig. 6:**
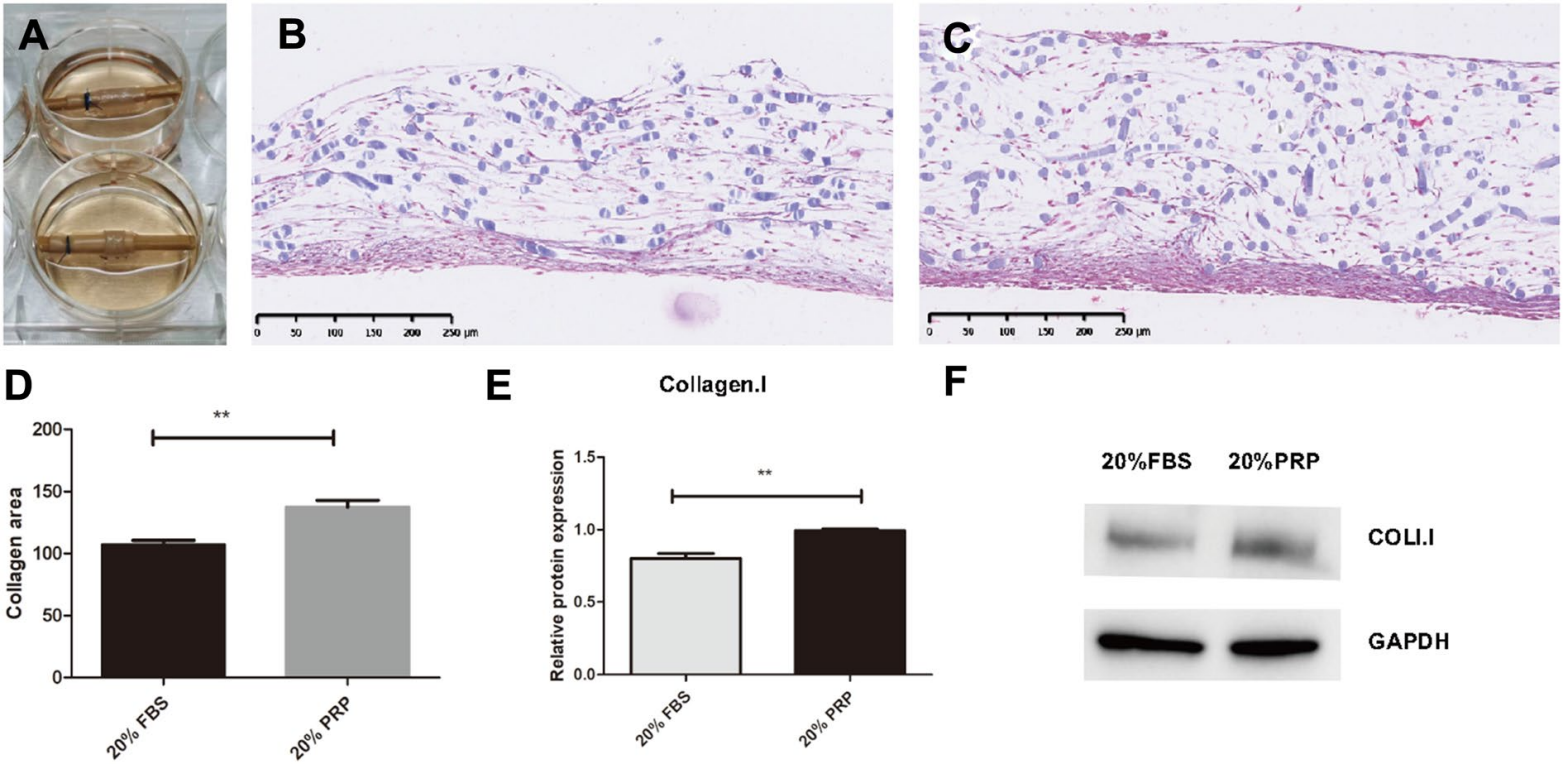
Three-dimensional culture results and gross plots, 3D culture for both treatments, plots of cultures after 4 weeks of incubation (**A**), plots of Masson staining and collagen staining for quantitative analysis of cultures (**B**–**D**), and WB assay for protein collagen I content and its grayscale value conversion (**E**, **F**). **p < 0.01 compared to the blank group, all scale bars = 250 µm

The cells of the experimental group and the control group were placed in 6-well plates for three-dimensional culture (Fig. 6A), and the cultures were removed after 4 weeks of static culture. The cultures were subjected to Masson staining and WB analysis of collagen content, and the results of Masson staining showed that the cells of the experimental group were more closely arranged, the collagen was stained blue, and the blue-stained collagen content of the experimental group was significantly higher than that of the control group, as seen by the naked eye. ImageJ statistics also confirmed this pattern, and the difference was statistically significant (Fig. 6B–D). WB results showed that the collagen content of the experimental group was significantly higher than that of the control group (Fig. 6E, F).

## Discussion

PRP is widely used in material-assisted applications [24]; however, its application in tissue-engineered vessels is rarely addressed. Does PRP form a connecting bridge between the seed cells of tissue-engineered vessels and the scaffold material, promoting the biological state of adhesion and secretion of SMCs on top of the scaffold material? We do not know. To address this question, we introduced PRP during the inoculation of smooth muscle cells into the scaffold and found that PRP could release the growth factors PDGF-BB and TGF-β continuously on the scaffold material for up to 30 days by detecting the content of growth factors in the culture medium. We investigated the effect of PRP on smooth muscle cell proliferation and migration by designing different experimental groups, determined the optimal concentration of PRP to use, and studied the adhesion behavior of smooth muscle cells after the addition of PRP and the secretion of collagen under three-dimensional culture conditions.

PRP has been used clinically for decades, and the preparation and distribution of PRP tend to be diverse [25]. The main reason is that PRP is rich in many growth factors [26, 27], among which PDGF-BB and TGF-β are closely related to the biological activity of cells [28, 29]. PDGF and TGF-β were found to significantly promote the proliferative activity of smooth muscle cells [30, 31], and Perrault R et al. showed that PDGF-BB could promote the conversion of smooth muscle cells to a secretory phenotype [32]. PDGF was also shown to promote the migration of smooth muscle cells by regulating the gene expression of MMP2 and MMP9 [33], although PRP contains a large number of growth factors. Although PRP contains a large amount of growth factors, the hydrogel formed after activation of PRP is unstable, and it is difficult to achieve stable release, so many researchers have used it together with other materials, such as PRP combined with sodium alginate for cartilage repair experiments [34], PRP combined with GelMA gel for 3D printing for rabbit cartilage repair experiments [11], or PRP combined with filament proteins using [35]. Although these methods effectively address the problem of PRP hydrogel stability, they are more cumbersome to perform. We added PRP mixed with smooth muscle cells directly to the surface of PGA material, and the electron microscopic results found that the fibrin filaments inside PRP evenly covered and filled the whole PGA fibers and fiber pores, and the adhesion of smooth muscle cells to PGA material because of PRP was not only due the adhesion of smooth muscle cells on PGA fibers but also due to the large number of adhesions between PGA pores. PRP promoted the adhesion of smooth muscle cells inside the PGA material not only by this physical action but also by mechanisms shown by the qPCR results, which showed that PRP promoted the expression of CTNNB1 and p-GSK-3β in smooth muscle cells, thus enhancing the adhesion function of smooth muscle cells and promoting the migration of smooth muscle cells through increased gene expression of MMP2 and MMP9. In addition, the results showed that PRP promoted the water uptake of PGA materials.

Collagen I is a major component of the extracellular matrix [36]. To verify that the addition of PRP can ultimately enhance extracellular matrix deposition in vascular tissue engineering, we cultured the experimental and control groups with PRP for 4 weeks and found that the vessels in the experimental group had more collagen deposition, and this result was fully confirmed in the WB experiments. In addition, the technique of repeated freeze‒thawing PRP to obtain growth factors is quite mature, and in the future, PRP can be obtained by processing frozen plasma for better application in tissue engineering vessel culture, which is convenient for future clinical applications [15]. Although the experiments showed that the addition of 20% PRP increased the adhesion function and migration of smooth muscle cells on top of PGA material and that the increase in collagen secretion after the addition of PRP was confirmed by three-dimensional culture, the specific mechanism of action remains to be confirmed for future application in tissue-engineered vascular culture.

## Conclusion

In this study, we used PRP mixed with smooth muscle cells and added nonwoven PGA material on top of the PRP before it formed a gel using a drop-in method. MMP9 expression was also increased. Three-dimensional culture resulted in more collagen deposition. Our study provides a convenient and effective way to promote good cell adhesion and low cell utilization in tissue-engineered vascular cultures in the future.

## Data Availability

Not applicable.

## Abbreviations

PRP: Plate rich plasma
FBS: Fetal bovine serum
SMC: Smooth muscle cell
ECM: Decellularized extracellular matrix
PGA: Polyglycolic acid
PLLA: Poly-l-lactic acid
PCL: Polycaprolactone
DMEM: Dulbecco’s Modified Eagle Medium
TGF-β: Transforming growth factor
PDGF-BB: Platelet-derived growth factor
COLI.1: Collagen type I
CNN: Calponin
SMTN: Smoothelin
MMP2: Matrix metalloproteinase 2
MMP9: Matrix metalloproteinase 9
CTNNB1: Recombinant human catenin beta-1
GSK3β: Glycogen synthase kinase 3β
GAPDH: Glyceraldehyde-3-phosphate dehydrogenase
